# Supplementary material for: National Early Warning Score (NEWS) Outperforms Quick Sepsis-Related Organ Failure (qSOFA) Score for Early Detection of Sepsis in the Emergency Department
Source: Antibiotics (Basel). 2022 Oct 31;11(11):1518. doi: 10.3390/antibiotics11111518 (PMC9686998; doi:10.3390/antibiotics11111518)
Supplement: Supplementary file 1 [file antibiotics-11-01518-s001.zip › antibiotics-1973314-supplementary.pdf]

Supplementary Table S1. Sensitivity and specificity of qSOFA and NEWS according to site of infection.

Site of infection		NEWS ≥ 5		qSOFA ≥ 2	
	**N**	**Sensitivity**	**Specificity**	**Sensitivity**	**Specificity**
Pulmonary	228/322	90 (86-94)	49 (38-59)	30 (24-37)	93 (85-97)
Urogenital	56/102	68 (54-80)	67 (52-80)	36 (23-50)	87 (74-95)
Gastrointestinal	32/67	72 (53-86)	83 (66-93)	34 (19-53)	97 (85-100)
Osteoarticular	15/17	73 (45-92)	100 (16-100)	40 (16-68)	100 (16-100)
Cutaneous/soft tissues	16/29	62 (35-85)	69 (39-91)	6 (0-30)	85 (55-98)
Systemic	5/6	100 (48-100)	100 (3-100)	80 (28-99)	100 (3-100)
Unknown	2/5	100 (16-100)	67 (9-99)	50 (1-99)	100 (29-100)
Overall	354/548	83 (79-87)	62 (55-69)	32 (27-37)	92 (87-95)

Supplementary Table S2. Sensitivity and specificity of qSOFA and NEWS according to age.

**Age (yr) range**		**NEWS ≥ 5**		**qSOFA ≥ 2**	
	**N**	**Sensitivity**	**Specificity**	**Sensitivity**	**Specificity**
18-44	6/20	100 (54-100)	57 (29-82)	33 (4-78)	93 (66-100)
45-54	14/29	93 (66-100)	67 (38-88)	21 (5-51)	87 (60-98)
55-64	29/59	83 (64-94)	47 (28-66)	31 (15-51)	93 (78-99)
65-74	60/96	90 (79-96)	47 (30-65)	33 (22-47)	97 (85-100)
75-84	85/161	80 (70-88)	57 (45-68)	27 (18-38)	91 (82-96)
85-above	106/191	88 (80-93)	59 (48-69)	42 (33-52)	86 (77-92)
All	300/556	86 (82-90)	55 (49-62)	34 (29-40)	90 (86-94)
